# Accessibility to Digital Mental Health Services among the General Public throughout COVID-19: Trajectories, Influencing Factors and Association with Long-Term Mental Health Symptoms

**DOI:** 10.3390/ijerph19063593

**Published:** 2022-03-17

**Authors:** Zheng-An Lu, Le Shi, Jian-Yu Que, Yong-Bo Zheng, Qian-Wen Wang, Wei-Jian Liu, Yue-Tong Huang, Xiao-Xing Liu, Kai Yuan, Wei Yan, Jie Shi, Yan-Ping Bao, Lin Lu

**Affiliations:** 1 Peking University Sixth Hospital, Peking University Institute of Mental Health, NHC Key Laboratory of Mental Health (Peking University), National Clinical Research Center for Mental Disorders (Peking University Sixth Hospital), Peking University, Beijing 100191, China; 1911210616@bjmu.edu.cn (Z.-A.L.); leshi@bjmu.edu.cn (L.S.); quejianyu@bjmu.edu.cn (J.-Y.Q.); yongbozheng@bjmu.edu.cn (Y.-B.Z.); qianwenwang@stu.pku.edu.cn (Q.-W.W.); weijian191954@stu.pku.edu.cn (W.-J.L.); yuetonghuang@stu.pku.edu.cn (Y.-T.H.); 1601111460@pku.edu.cn (X.-X.L.); yuankai@pku.edu.cn (K.Y.); weiyan@bjmu.edu.cn (W.Y.); 2 Peking-Tsinghua Center for Life Sciences and PKU-IDG/McGovern Institute for Brain Research, Beijing 100871, China; 3 National Institute on Drug Dependence and Beijing Key Laboratory of Drug Dependence, Peking University, Beijing 100191, China; shijie@bjmu.edu.cn

**Keywords:** digital mental health services, COVID-19, accessibility, trajectory

## Abstract

Digital mental health services (DMHSs) have great potential for mitigating the mental health burden related to COVID-19, but public accessibility (ease of acquiring services when needed) to DMHSs during the pandemic is largely unknown. Accessibility to DMHSs was tracked longitudinally among a nationwide sample of 18,804 adults in China from before to one year after COVID-19 outbreak. Unconditional and conditional latent growth curve models and latent growth mixture models were fitted to explore the overall growth trend, influencing factors, and latent trajectory classes of accessibility to DMHSs throughout COVID-19. Generalized estimating equation models and generalized linear mixed models were employed to explore the association between accessibility to DMHSs and long-term mental health symptoms. We found that people generally reported increased difficulty in accessing DMHSs from before to one year after COVID-19 outbreak. Males, youngsters, individuals with low socioeconomic status, and individuals greatly affected by COVID-19 reported greater difficulty in accessing DMHSs. Four DMHS accessibility trajectory classes were identified: “lowest–great increase” (6.3%), “moderate low–slight increase” (44.4%), “moderate high–slight decrease” (18.1%) and “highest–great decrease” (31.2%). Trajectory classes reporting greater difficulty in accessing DMHSs were at higher risk for long-term mental symptoms. In conclusion, an overall increase in difficulty in accessing DMHSs is observed throughout COVID-19, and heterogeneity exists in DMHS accessibility trajectories. Our results suggest that easy access to DMHSs should be consistently facilitated. Moreover, access gaps should be reduced across demographic groups, and target populations for service allocation should alter as the pandemic evolves.

## 1. Introduction

The COVID-19 pandemic has spread throughout the globe and caused over 440 million infected cases and 5.9 million deaths (World Health Organization. COVID-19 Dashboard, accessed on 7 March 2022). The resulting pervasive pandemic control measures have put society under substantial mental health strain. About one-third of the global population reported mental health symptoms during COVID-19 [1,2,3,4]. Given the profound mental health repercussions of COVID-19, appropriate sources of mental health support are urgently called for to mitigate mental health impacts caused by the pandemic.

As high transmission rate, strict traffic control, and social distancing measures preclude large-scale delivery of face-to-face psychological interventions during COVID-19, digital mental health services (DMHSs) are considered desirable alternatives due to their high feasibility, safety, and effectiveness [5,6]. DMHSs have been widely provided during COVID-19 as an indispensable part of psychological crisis intervention in various forms: digital educational programs, digital psychological counseling, digital mental health knowledge, as well as digital instructions and guidelines for mental health protection [5,7]. These services have been highly valued for their role in mental health promotion during this period [5,6,8].

Despite these strong merits of DMHSs, their full and efficient utilization should be ensured to promote public mental health. Additionally, given the limited space of online platforms and digital resources, the major aim for DMHS delivery is to ensure those in actual need can easily acquire DMHSs instead of simply increasing general DMHS usage. Therefore, accessibility, defined as ease of acquiring services when needed, should be investigated for DMHSs to guide precise service delivery [9,10].

Based on the theoretical domains framework, determinants for medical service accessibility can be categorized into several domains, ranging from social and environmental resources to individual skills and motivations [11]. More specifically, the digital health equity framework (DHEF) summarized determinants for digital health service accessibility as several domains, including access to digital media, digital literacy, and integration of digital resources into community [12]. Currently, there are two measures of accessibility: objective accessibility and perceived accessibility. Conventional objective measures mainly focus on external aspects of service availability, such as quantity and coverage, which cover only a small fraction of accessibility determinants in the complete framework [13]. However, perceived accessibility, defined as a “subjective rating of ease of access to services or resources”, can reflect the mixture of objective service availability, individual-level abilities to use services, as well as other social and environmental aspects, thus covering most aspects in the theoretical framework [13,14]. Therefore, perceived accessibility is considered as a more comprehensive accessibility measure and is frequently identified as key antecedent for full public service utilization [13,14]. The current research mainly focused on perceived accessibility to DMHSs during COVID-19.

Moreover, since accessibility to DMHSs might change throughout COVID-19 due to fluctuating service supplies and social encouragement, trajectory of accessibility to DMHSs should be captured to provide time-specific directions for service delivery [5,15,16,17]. Additionally, since a digital divide has long been observed in low- and middle-income countries, populations with special difficulties in accessing DMHSs should be identified during COVID-19 [6]. Whether COVID-19 can further widen the existing digital divide is another important question to address. Further, if heterogeneity exists in the DMHS accessibility trajectories, identifying demographic features of classes with distinct evolving patterns of DMHS accessibility can be highly informative for precise delivery. Delving into the in-depth information related to DMHS accessibility requires advanced modeling methods.

Trajectory modeling approaches have been frequently employed to describe the evolution of self-rating measures, mental symptoms, behaviors, and biomarkers [18,19]. These methods model trajectories by constructing and estimating latent intercept and slope variables based on a set of observable measures collected at different time points. They can thus separately describe the initial level and changing trend of a given observable measure and identify influencing factors [18,19]. Therefore, trajectory modeling approaches can not only identify the demographic gaps of a measure but also ascertain whether the gaps are widened or narrowed over time. Additionally, based on inter-individual relationships, trajectory modeling approaches can classify individuals into different trajectory pattern classes by involving an additional latent categorical variable. Therefore, these methods demonstrate strong merits in exploring trajectory heterogeneity [18]. Further, leveraging full information maximum likelihood (FIML) estimation, trajectory modeling approaches can mitigate the power loss due to missing data in longitudinal analyses [20,21]. Based on these merits, we adopted trajectory modeling approaches to describe DMHS accessibility evolution in this research. The latent growth curve model (LGCM) allows the examination of the overall growth trend of accessibility to DMHSs [22]. A conditional LGCM can help identify demographic gaps of DMHS accessibility [16]. The latent growth mixture model (LGMM) can divide the whole population into several classes following distinct evolving patterns of accessibility [23,24].

In addition, although some researchers suggest the potential of DMHSs in mitigating mental health burden during COVID-19, few existing studies focus on population-level effects of DMHS provision [25,26]. Policymakers and DMHS providers are eager to ascertain the actual social benefits of facilitating DMHS access. Investigation of association between DMHS accessibility trajectories and long-term mental health symptoms can provide some insights.

Therefore, based on longitudinal data from a nationwide sample in China, the current research has the following four aims: (1) to estimate the overall growth trend of accessibility to DMHSs throughout COVID-19; (2) to identify populations with greater difficulty in accessing DMHSs throughout COVID-19; (3) to categorize individuals into distinct classes based on DMHS accessibility trajectories and identify the specific demographic features of each class; (4) to explore the association between accessibility to DMHSs and long-term mental health symptoms.

## 2. Methods

### 2.1. Procedures and Participants

We conducted a longitudinal observational study in which participants were recruited from the Chinese website Joybuy. Joybuy is a large ecommerce and information clustering website that provides online services with 0.44 billion active users from all 34 provinces in China. We selected Joybuy as our survey platform because of its wide usage in China. The members of Joybuy are generally young and highly educated. Membership is acquired by online registration with an annual fee [3,4]. Data were collected three times since the outbreak of COVID-19. Survey 1 was conducted during the initial peak of COVID-19 (28 February 2020 to 11 March 2020). Survey 2 was conducted in the aftermath of the initial COVID-19 peak (8 July 2020 to 8 August 2020), when the initial peak had been basically controlled but sporadic cases were still seen. Survey 3 was conducted during the post-COVID-19 period (29 January 2021 to 26 April 2021). During Survey 1, all registered members were allowed to click on a link to participate in the survey until the total sample represented all 34 province-level regions in China, as detailed elsewhere [3]. During Survey 2 and Survey 3, two approaches were adopted to recruit participants. Firstly, we adopted a targeted approach, in which survey links were sent via the message platform of Joybuy to all participants who responded to at least one previous survey. On the other hand, to recruit new participants, we adopted an untargeted approach, in which we put links of Survey 2 and Survey 3 on the Joybuy website, allowing new participants to voluntarily click on them. Shopping vouchers were offered to those who completed the surveys.

**Figure 1:**
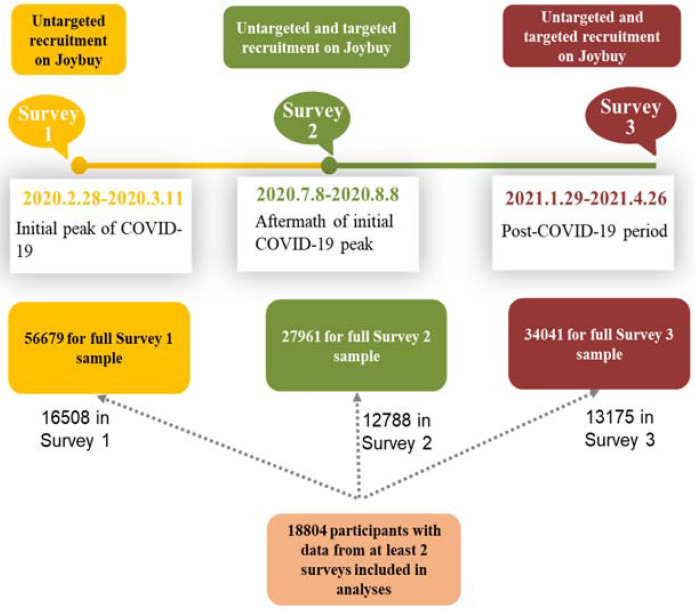
Flow graph for participants recruitment in three surveys.

All participants were registered members of Joybuy. During Survey 1, a total of 56,679 adults providing valid age information were included, as detailed elsewhere [3]. The final longitudinal sample for analyses comprised 18,804 adults with data from at least two of the three surveys, among whom data were available for 16,508 from Survey 1, 12,788 from Survey 2, and 13,175 from Survey 3 (Figure 1).

### 2.2. Measures

In all three surveys, we measured mental health symptoms including depression, anxiety, and insomnia and queried participants regarding demographics, epidemic-related conditions, accessibility, and actual usage of DMHSs using self-designed questionnaires. Detailed contents of the questionnaires were provided elsewhere [3,4]. Anonymousness, confidentiality, and voluntariness were ensured in all surveys.

Accessibility to DMHSs was measured in three surveys with a self-reported item on a visual analogue scale (VAS): Please rate your difficulty in acquiring digital mental health services (information about psychological interventions and psychological knowledge provided via digital media including TV, Internet, and mobile phones) when needed at present: 0 (highest accessibility, very easy/not difficult at all to access digital mental health services) to 10 (lowest accessibility, not easy at all/very difficult to access digital mental health services). Participants were requested to self-report the accessibility before COVID-19 in Survey 1. Perceived accessibility is usually measured with a visual analogue scale (VAS) [27,28].

Usage of DMHSs was measured in three surveys using the self-report item: Have you accessed digital mental health services (information about psychological interventions and psychological knowledge provided via digital media including TV, Internet, and mobile phones) in the recent three months? The answers included “Yes” and “No”. Participants were asked to self-report DMHS usage before COVID-19 outbreak in Survey 1.

In both the baseline and follow-up surveys, Chinese versions of the Patient Health Questionnaire-9 (PHQ-9), Generalized Anxiety Disorder-7 (GAD-7), and Insomnia Severity Index (ISI) were used to measure symptoms of depression, anxiety, and insomnia, respectively. We used cut-off scores of 5, 5, and 8 to categorize participants as depressed, anxious, and having insomnia symptoms [29,30,31]. Participants with depression, anxiety, or insomnia were categorized as having mental health symptoms.

### 2.3. Statistical Analyses

**Figure 2:**
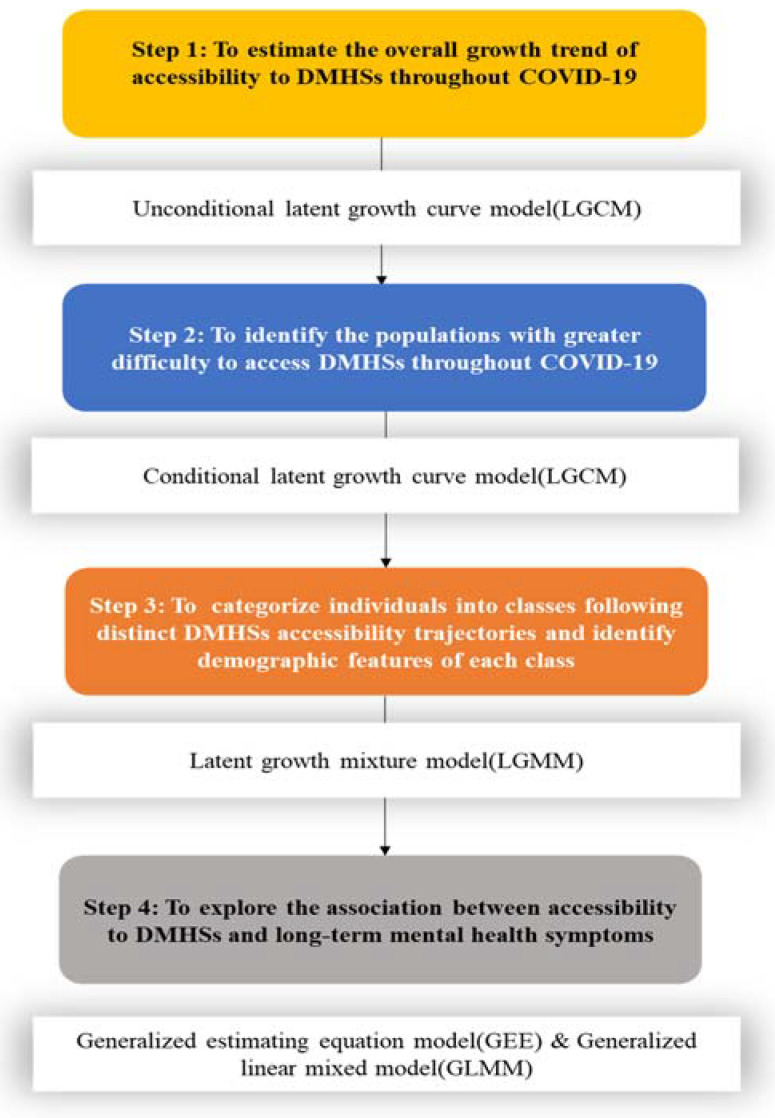
Procedures for statistical analyses.

Descriptive statistics were used to present the baseline demographics and epidemic-related characteristics. Next, to investigate trajectories and influencing factors of accessibility to DMHSs, we analyzed the data in four steps. Figure 2 illustrates the procedures for statistical analyses in the current research.

In the first step, the overall changing trend of accessibility was explored by fitting an unconditional LGCM, in which the outcome variables were the self-reported accessibility scores before COVID-19 (measured in Survey 1), during initial peak (measured in Survey 1), after initial peak (measured in Survey 2), and in the post-COVID-19 period (measured in Survey 3). We tested an LGCM with two types of growth factors (intercept-only and linear slope). For the LGCM with a linear slope, we further tested models with fixed (coded as 0, 1, 6, and 13 for the four time points) or free slope factor loadings. An LGCM with quadratic slope was also fitted but rejected due to poor fitting statistics. The final optimal model was selected based on comparative fit index (CFI), chi-squared, standardized root-mean-square residual (SRMR), and the root mean square error of approximation (RMSEA) statistics. Smaller values for chi-squared, RMSEA, and SRMR and larger values for CFI suggest a better fit [32]. In the unconditional LGCM model, the mean for the intercept reflects the average initial accessibility level (before COVID-19), and the mean for slope reflects the average change in accessibility.

In the second step, to identify populations with greater difficulty in accessing DMHSs, we fitted a conditional LGCM by adding influencing factors into the optimal LGCM in step 1, so that the intercept and slope for accessibility could be regressed on these factors. All influencing factors considered were categorized into 3 groups: demographic factors, COVID-19-infection-related factors, and factors related to secondary social repercussions. Demographic factors included gender, age groups, living area, educational level, marital status, and income level. COVID-19-infection-related factors included being COVID-19 patients or close contacts and engaging in COVID-19-related work. Factors related to secondary social repercussions included living in places severely affected by COVID-19, quarantine experiences, increases in workload, unemployment due to COVID-19, and seeking psychological consultation after COVID-19. In the conditional LGCM model, the effect values for the intercept reflect the effects of influencing factors on initial DMHS accessibility level (before COVID-19), while effect values for slope reflect the effects of influencing factors on longitudinal change in DMHS accessibility throughout COVID-19.

In the third step, we applied a linear slope LGMM to identify latent trajectory classes of DMHS accessibility, so that individuals can be categorized into classes following distinct DMHS accessibility trajectory patterns. We gradually increased the number of latent trajectory classes from 1 to 7 and determined the optimal number of classes based on parsimony, interpretability, sufficient individuals in each class, Akaike information criterion (AIC) [33], Bayesian information criterion (BIC) [34], adjusted BIC (aBIC) [35], Lo–Mendell–Rubin likelihood ratio test (LMR-LRT) [36], and entropy values [36]. Classes with individuals accounting for <5% of the total sample was not considered, since they might appear due to class over-extraction [37]. Lower BIC, aBIC, and AIC values indicate a better fit [33,34,35]. A significant *p* value in LMR-LRT suggests a better fit of model with k trajectory classes compared with model with k-1 trajectory classes [36]. Entropy characterizes quality of classification on a 0 to 1 scale, with values closer to 1 indicating a more accurate classification and an entropy value of 0.60 indicating about 20% classification errors [36]. In all steps above, missing data were handled by full information maximum likelihood estimation, based on the assumption that missingness was at random [38]. After determining the optimal number of latent classes, all individuals were assigned to the latent trajectory class based on posterior probability. Descriptive statistics were employed to present demographic and epidemic-related characteristics of the four trajectory classes.

In the fourth step, to investigate the association between accessibility trajectory class membership and long-term mental health outcomes, we performed two analyses. In the first analysis, we treated mental health symptoms as categorical variables and investigated the association between accessibility trajectory class membership and long-term positives of mental health symptoms. We fitted four generalized estimating equation (GEE) models with binomial distribution and autoregressive covariance structures. In the four GEE models, outcome variables were categorical status (yes/no) for depression, anxiety, insomnia, and any mental health symptoms, while the independent variables were accessibility latent class membership, with survey order as a within-subject effect and participant ID number as a covariate factor. Covariates were adjusted for in all four GEE models. In the second analysis, we treated mental health symptoms as continuous variables and investigated the association between accessibility trajectory class membership and long-term mental health symptom scores. We fitted three generalized linear mixed models (GLMMs) with random within-subject intercepts and autoregressive covariance structures. In the three GLMMs, outcome variables were the continuous PHQ-9, GAD-7, and ISI scores, and the independent variables were accessibility latent class membership and interaction terms with time (coded as 0, 5, and 12 in three surveys) as fixed effects. We also adjusted for the fixed effects for covariates and their interaction terms with time in all three GLMMs.

To further validate our results with more objective measures, we performed supplementary analyses on actual DMHS usage throughout COVID-19. Proportions of individuals reporting digital DMHS usage in three surveys were presented. 

The level of significance was set to two-sided *p* < 0.05. All the statistical analyses were performed with SPSS 22 software (SPSS, Chicago, IL, USA), Mplus 8.3 (Muthen & Muthen, Los Angeles, CA, USA), and R version 4.0.3.

## 3. Results

### 3.1. Demographic Characteristics of the Longitudinal Sample

Table 1 presents demographic characteristics of the total sample. Of the 18,804 participants, the mean (SD) age was 36.6 (8.2), and 8558 (45.5%) were male, 17,599 (93.6%) lived in urban areas, 15,489 (82.4%) had a college school or higher educational level, 14,783 (78.6%) were married, and 4186 (22.3%) had family monthly income lower than CNY 5000.

### 3.2. Trajectory of Accessibility to DMHSs from Before to One Year after COVID-19 Outbreak

**Figure 3:**
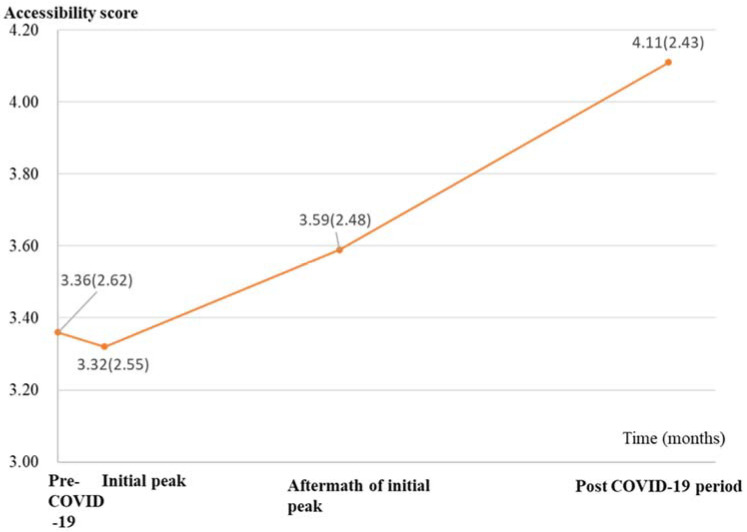
Trajectory of accessibility to DMHSs from before to one year after COVID-19 outbreak. Higher scores indicate lower accessibility level (more difficult to access DMHSs). Raw mean accessibility scores (SD) are presented at each time point.

The linear slope model with free slope factor loadings (CFI = 0.95, chi-squared value = 434.14, RMSEA = 0.09, SRMR = 0.04) indicated a better fit compared with the intercept only model (CFI = 0.42, chi-squared value = 4941.16, RMSEA = 0.18, SRMR = 0.23) and the linear slope model with fixed slope factor loadings (CFI = 0.90, chi-squared value = 901.77, RMSEA = 0.10, SRMR = 0.10). Therefore, the linear slope model with free slope factor loadings was selected as the optimal model, in which the estimated mean (SE) for the intercept was 3.31 (0.02) (*p* < 0.001), and the estimated mean (SE) for slope was 0.13 (0.01) (*p* < 0.001), indicating a significant overall increase in difficulty in accessing DMHSs (Figure 3).

### 3.3. Influencing Factors of Accessibility to DMHSs throughout COVID-19

Table 2 presents the results from the conditional LGCMs. Males, youngsters, individuals greatly affected by COVID-19 (i.e., individuals engaging in COVID-19-related work, living in places severely affected by COVID-19, experiencing quarantine, increases in workload, unemployment, or seeking psychological intervention after COVID-19) reported greater difficulty in accessing DMHSs throughout COVID-19. Individuals with low socioeconomic status (i.e., rural residence and low income level) also reported generally greater difficulty, though the effects were non-significant. Moreover, compared with others, males and individuals greatly affected by COVID-19 (i.e., COVID-19 patients or close contacts, individuals engaging in COVID-19-related work, suffering from increases in workload, experiencing unemployment, or seeking psychological intervention after COVID-19) demonstrated a steeper increase (or milder decrease) in difficulty in accessing DMHSs from before to one year after COVID-19 outbreak, suggesting these accessibility gaps were widened throughout COVID-19.

### 3.4. Latent Trajectory Classes of Accessibility to DMHSs from Before to One Year after COVID-19 Outbreak

**Figure 4:**
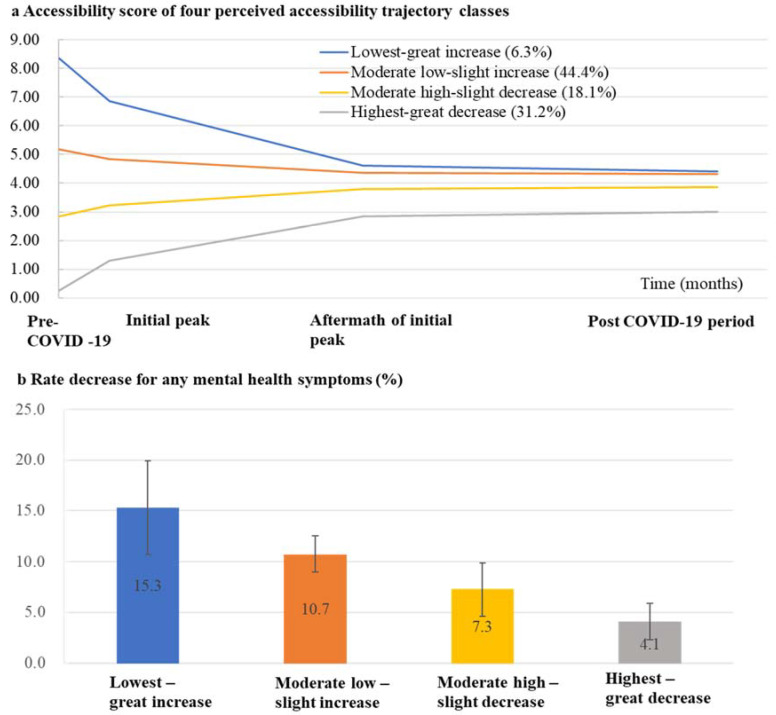
(**a**) Latent trajectory classes of accessibility to DMHSs from before to one year after COVID-19 outbreak from the best fitting four-class LGMM. Higher scores indicate lower accessibility level (more difficult to access DMHSs). (**b**) Rate decrease for any mental health symptoms from initial COVID-19 peak (Survey 1) to post-COVID-19 period (Survey 3) stratified by four accessibility trajectory classes. Rate decrease was calculated by subtracting rate of any mental health problems in Survey 3 from the rate in Survey 1. Mental health symptoms are defined as depression, anxiety, or insomnia. Error bars indicate 95% confidence intervals.

Table 3 presents model fitting statistics for LGMMs with one to seven trajectory classes. Compared with models with one to three classes, the four-class model had lower AIC, BIC, and aBIC values, greater entropy, and a significant *p* value for LMR-LRT, suggesting its better fit and more accurate classification. In addition, the four-class model had sufficient individuals in each latent class (smallest latent class proportion: 6.3%) and greater parsimony. However, in models with five to seven classes, the smallest latent classes accounted for a proportion < 5%. Since decreases in AIC, BIC, and aBIC decreased when number of classes increased from four to seven, we selected the four-class model as the optimal model, as shown in Figure 4a.

In the four-class final model, we identified two classes showing lower average accessibility level (greater average difficulty in accessing DMHSs) but an increasing accessibility trend (longitudinal decrease in difficulty in accessing DMHSs), which were named “low–increase” accessibility pattern classes. The two classes were labeled as “lowest–great increase” accessibility class (mean (SE) for intercept: 8.35 (0.19), *p* < 0.001; mean (SE) for slope: −1.50 (0.08), *p* < 0.001) and “moderate low–slight increase” accessibility class (mean (SE) for intercept: 5.18 (0.07), *p* < 0.001; mean (SE) for slope: −0.33 (0.03), *p* < 0.001) and respectively accounted for 6.3% and 44.4% of the overall sample. We identified another two classes showing higher average accessibility level (lower average difficulty in accessing DMHSs) but a decreasing accessibility trend (longitudinal increase in difficulty in accessing DMHSs), which were named “high–decrease” accessibility pattern classes. The two classes were labeled as “moderate high–slight decrease” accessibility class (mean (SE) for intercept: 2.83 (0.05), *p* < 0.001; mean (SE) for slope: 0.39 (0.02), *p* < 0.001) and “highest–great decrease” accessibility class (mean (SE) for intercept: 0.27 (0.01), *p* < 0.001; mean (SE) for slope: 1.04 (0.02), *p* < 0.001) and respectively accounted for 18.1% and 31.2% of the overall sample.

Table 4 and Table S1 show demographic and epidemic-related characteristics of the four latent trajectory classes. The two “low–increase” accessibility pattern classes featured higher proportion of males, youngsters, individuals with low socioeconomic status (i.e., low education, low income, and rural residence), and individuals greatly affected by COVID-19 (i.e., COVID-19 patients or close contacts, individuals engaging in COVID-19-related work, with quarantine experiences, living in places severely affected by COVID-19, suffering from increases in workload, experiencing unemployment, or seeking psychological consultation after COVID-19). The two “high–decrease” accessibility pattern classes featured higher proportion of females, mid-aged or elderly people, and individuals with high socioeconomic status (i.e., high education, high income, and urban residence).

### 3.5. Association between Trajectory Class Membership of Accessibility to DMHSs and Long-Term Mental Health Symptoms

Table 5 and Table 6 and Figure 4b show the association between accessibility trajectory class and long-term mental health symptoms. We found risk for developing any long-term mental health symptoms decreased accordingly as the average difficulty in accessing DMHSs decreased from the “lowest–great increase” accessibility trajectory class to the “highest–great decrease” accessibility trajectory class (“lowest–great increase” class: adjusted odds ratio (95% CI), 2.75 [2.47–3.05]; “moderate low–slight increase” class: 2.56 [2.41–2.72]; “moderate high–slight decrease” class: 1.79 [1.66–1.93]; “highest–great decrease” class: reference). Similar trend was also found in GEE analysis for single symptoms (Table 5) and GLMM analyses with continuous symptom scores as outcome variables (Table 6).

Moreover, we found trajectory classes reporting greater reduction in difficulty in accessing DMHSs over time experienced more substantial alleviation in mental health symptoms throughout COVID-19: “lowest–great increase” accessibility class demonstrated highest rate decrease in any mental health symptoms (15.3%), which was followed by “moderate low–slight increase” (10.7%), “moderate high–slight decrease” (7.3%), and “highest–great decrease” accessibility class (4.1%) (Figure 4b and Table 5). The finding was confirmed by the significant effects for the trajectory class × time interaction terms in GLMMs (Table 6).

### 3.6. Supplementary Analyses of DMHS Usage throuhout COVID-19

Despite a mild increase from before to initial peak, proportion of DMHS usage remarkably dropped from initial peak to post-COVID-19 period (Figure S1).

## 4. Discussion

This is the first study to investigate public accessibility to DMHSs during COVID-19. We found that people generally reported increased difficulty in accessing DMHSs from before to one year after COVID-19 outbreak. Males, youngsters, individuals with low socioeconomic status, and individuals greatly affected by COVID-19 reported greater difficulty in accessing DMHSs, and the gap was further widened between individuals greatly affected by COVID-19 and others as COVID-19 evolved. Individuals reporting greater difficulty in accessing DMHSs showed higher risk of long-term mental health symptoms. Our findings could provide reference for DMHS allocation and delivery throughout COVID-19.

Previous studies suggested that accessibility to digital services depended on the following four aspects: (1) supplies of digital services; (2) individual access to digital media (i.e., computers, mobile phones, and TVs) and technologies (i.e., apps and Internet); (3) literacy and accessibility (i.e., experience in digital technologies, relevant knowledge, and initiative to seek services); (4) appropriate environment for DMHS uses (i.e., social encouragement, guidance and support) [39,40,41]. Therefore, accessibility trajectories might be influenced by alterations in either of the four aspects.

### 4.1. Trajectory of Accessibility to DMHSs during COVID-19

We found that people generally reported increased difficulty in accessing DMHSs from before to one year after COVID-19 outbreak. Interestingly, we observed that actual usage of DMHSs accordingly decreased during the same period, further validating our results. The slight but significant increase in accessibility (*p* from paired-samples *t* test = 0.02) from before COVID-19 to the initial COVID-19 peak might be attributable to rapid provision of abundant digital mental health resources during initial peak [5]. However, it is noteworthy that accessibility experienced a dramatic decrease after initial peak. One possible explanation is that provision of DMHSs decreased when the pandemic was largely under control and media focus was shifted away from mental health. It is also possible that the general public had more time and motivation during initial peak to use digital devices due to more flexible home-working styles [42,43,44]. Further, there was more social encouragement for DMHS usage during initial peak [15]. Since about 30% of people still suffered from mental health symptoms after initial peak, continuous increase in difficulty in accessing DMHSs reflects a gap between demand and access to DMHSs in the late COVID-19 phase [4,16,17,45,46]. Therefore, easy access to DMHSs should be continuously facilitated throughout COVID-19.

### 4.2. Latent Trajectory Classes of Accessibility to DMHSs during COVID-19

We identified four latent trajectory classes of accessibility to DMHSs. Distinct average accessibility level in four classes echoed with a remarkable accessibility gap before COVID-19, suggesting the need to promote equality in access to DMHSs [47]. Despite the overall increase in difficulty in accessing DMHSs, nearly half of the participants reported longitudinal decrease in difficulty in accessing DMHSs. Our further analyses showed that trajectory classes with difficulty decrease featured a higher proportion of males, youngsters, and individuals with low socioeconomic status. These populations tended to experience greater work burden before COVID-19. The shift to more flexible home-working styles during lockdown might have provided them with more time and motivation to search for digital services [42,43]. Trajectory classes with difficulty decrease also featured higher proportion of individuals greatly affected by COVID-19, who might be most strongly affected during initial peak and thus encounter the greatest accessing difficulty at that stage. The findings indicate that DMHS delivery strategies should be tailored to different populations. Additionally, although over 60% individuals followed relatively stable changing pattern, nearly 40% participants reported substantial change in accessibility, indicating key populations for DMHS delivery should alter as the pandemic evolves.

### 4.3. Influencing Factors of Accessibility to DMHSs throughout COVID-19

We found males, youngsters, and individuals greatly affected by COVID-19 (i.e., COVID-19 patients or close contacts, individuals engaging in COVID-19-related work, living in places severely affected by COVID-19, experiencing quarantine, increases in workload, unemployment, or seeking psychological intervention after COVID-19) reported greater difficulty in accessing DMHSs throughout COVID-19. Individuals with low socioeconomic status (i.e., rural residence and low income level) also demonstrated generally greater difficulty in accessing DMHSs.

Males and youngsters were previously found to show less interest in health associated topics, thus they had lower literacy in mental health and lack of motivation to seek relevant services [40,48,49,50]. However, females were more likely to perceive their psychological needs and motivated to seek psychological help [51]. Mid-aged or elderly people were at greater risk for health-related problems, and thus showed higher health awareness and more motivation to seek health services [40,50].

As for individuals greatly affected by COVID-19, quarantine and financial strain experienced by them might lead to limited access to digital media [52]. The substantial workloads and fear of infection among high-risk workers could lead to emotional exhaustion and stress, decreasing their motivation and ability to seek DMHSs [53,54,55]. Moreover, we found that as COVID-19 evolved, the accessibility gap between individuals greatly affected by COVID-19 and others was widened, indicating easy access to DMHSs should be especially ensured among these vulnerable populations. The discrepancy in accessibility between high and low socioeconomic groups could be due to uneven access to digital media or technologies, different literacy and digital skills, as well as different level of social supports [39,56,57,58,59].

### 4.4. Association between Trajectory Class of Accessibility to DMHSs and Long-Term Mental Health Symptoms during COVID-19

Individuals with greater difficulty in accessing DMHSs demonstrated higher long-term risk for mental health symptoms, and reduction in DMHS access difficulty is predictive of more substantial amelioration in mental health symptoms. Similar associations were observed before COVID-19 [60]. Our results suggest that facilitating easy access to DMHSs may have the potential to mitigate mental health symptoms, in agreement with other studies [25,26]. However, we cannot rule out possibility that mental health symptoms may impair cognitive functions thus posing barriers to service access [47]. Therefore, more studies are called for to further ascertain the causal relationships between accessibility to DMHSs and long-term mental health symptoms.

## 5. Strengths and Limitations

To the best of our knowledge, this study was the first to focus on public accessibility to DMHSs during COVID-19. Since a digital divide has long been observed globally, and health resources have been increasingly provided via digital media after COVID-19, our research can offer information of a global interest on whether and how the digital exclusion will impact mental health inequality during the pandemic period [6,61]. The strengths of the study also include its large sample size and timeliness. We employed trajectory modeling approaches, offering a novel perspective in exploring digital service accessibility.

There are several limitations. First, accessibility was measured with a self-reported item, which might involve biases. However, according to our supplementary analyses, actual DMHS usage decreased accordingly as DMHS accessibility decreased throughout COVID-19. The findings indicate that DMHS accessibility in our research can at least partially reflect actual DMHS access. Future studies employing objective accessibility measures are required to produce more tenable results. Second, the current study did not involve theory-driven research based on an empirical theoretical framework; thus, the key determinant in reducing the DMHS accessibility gap cannot be determined. A recent theory-driven study based on a small sample has partially addressed the question by identifying insufficient digital or language skills as the major barrier blocking DMHS access during COVID-19 [12]. Our research findings can complement this work. Future relevant studies should be developed based on existing theoretical frameworks. Third, the sample could have been biased in its population structure in some demographic dimensions, including age, regions, and educational levels, due to the online recruitment strategy. Future analyses based on more representative samples are essential. Fourth, the study had a relatively low follow-up rate that could have involved bias in trajectory modeling. However, since we managed to acquire a large sample, and demographic characteristics did not differ much between our longitudinal sample and baseline full sample (Table S2), we believed the bias would not largely affect the robustness of our results. Additionally, this research was based on a nationwide sample, so relevant studies in other countries are required to replicate our findings. Fifth, we could not ascertain the causal relationship between DMHS accessibility and long-term mental health symptoms. Cohort studies are required to validate the actual effects of DMHSs during COVID-19. Consequently, future relevant studies should be developed on empirical theories, adopt objective tools for accessibility, and base analyses on more representative samples. Studies conducted in other countries are also welcomed.

## 6. Conclusions and Implications

The current research presents the following four conclusions: (1) People generally reported increased difficulty in accessing DMHSs from before to one year after COVID-19 outbreak; (2) males, youngsters, individuals with low socioeconomic status, and individuals greatly affected by COVID-19 reported greater difficulty in accessing DMHSs during COVID-19; (3) heterogeneity existed in DMHS accessibility trajectories; (4) ease of access to DMHSs was predictive of lower risk for long-term mental health symptoms throughout COVID-19, suggesting the probable social benefits of facilitating DMHS access.

Implications of the study include: (1) Easy access to DMHSs should be continuously facilitated throughout COVID-19; (2) accessibility gaps should be reduced between low and high socioeconomic groups, males and females, as well as youngsters and the elderly, and easy access to DMHSs should be particularly ensured among individuals greatly affected by COVID-19; (3) accessibility to DMHSs should be consistently looked out for throughout COVID-19, and delivery strategies should be tailored to different populations. We believe our findings can provide valuable information for DMHS delivery during pandemics.

## Figures and Tables

**Table 1:** Demographic and epidemic-related characteristics of the longitudinal sample.

Factors	No. (%)/Mean (SD)
Overall	18,804 (100.0)
Gender	
Male	8558 (45.5)
Female	10,246 (54.5)
Mean for age (SD)	36.6 (8.2)
Age group (years)	
18–39	12,364 (65.8)
≥40	6440 (34.2)
Living area	
Urban	17,599 (93.6)
Rural	1205 (6.4)
Educational level	
College school or higher	15,489 (82.4)
Lower than college school	3315 (17.6)
Marital status	
Married	14,783 (78.6)
Unmarried	4021 (21.4)
Income level (CNY)	
0–4999	4186 (22.3)
≥5000	14,618 (77.7)
History of chronic diseases	
Yes	1201 (6.4)
Unknown/no	17,603 (93.6)
History of mental disorders	
Yes	122 (0.6)
Unknown/no	18,682 (99.4)
Family history of mental disorders	
Yes	235 (1.2)
Unknown/no	18,569 (98.8)

**Table 2:** Influencing factors of the intercept and slope of perceived accessibility to DMHSs from the conditional latent growth curve model.

Influencing Factors of the Intercept	B (SE)	*p* Value
Gender: male (vs. female)	0.11 (0.04)	0.008
Age group: 18–39 (vs. ≥40)	0.34 (0.04)	<0.001
Living area: urban (vs. rural)	−0.13 (0.08)	0.12
Educational level: college school or higher (vs. lower than college school)	0.07 (0.06)	0.24
Marital status: married (vs. unmarried)	−0.01 (0.05)	0.87
Family monthly income: 0–4999 (vs. ≥5000)	0.09 (0.05)	0.06
COVID-19 patients or close contacts: yes (vs. no)	0.06 (0.17)	0.72
Engaging in COVID-19-related work: yes (vs. no)	0.12 (0.04)	0.005
Living in places severely affected by COVID-19: yes (vs. no)	0.19 (0.05)	<0.001
Quarantine: yes (vs. no)	0.39 (0.04)	<0.001
Increases in workload due to COVID-19: yes (vs. no)	0.49 (0.04)	<0.001
Unemployment due to COVID-19: yes (vs. no)	0.43 (0.06)	<0.001
Seeking psychological intervention: yes (vs. no)	0.74 (0.06)	<0.001
**Influencing Factors of the Slope**	**B (SE)**	***p* Value**
Gender: male (vs. female)	0.05 (0.01)	<0.001
Age group: 18–39 (vs. ≥40)	0.004 (0.014)	0.77
Living area: urban (vs. rural)	−0.02 (0.03)	0.42
Educational level: college school or higher (vs. lower than college school)	−0.02 (0.02)	0.40
Marital status: married (vs. unmarried)	−0.04 (0.02)	0.004
Family monthly income: 0–4999 (vs. ≥5000)	0.03 (0.02)	0.05
COVID-19 patients or close contacts: yes (vs. no)	0.20 (0.05)	<0.001
Engaging in COVID-19-related work: yes (vs. no)	0.08 (0.01)	<0.001
Living in places severely affected by COVID-19: yes (vs. no)	0.02 (0.02)	0.13
Quarantine: yes (vs. no)	−0.002 (0.014)	0.90
Increases in workload due to COVID-19: yes (vs. no)	0.05 (0.01)	<0.001
Unemployment due to COVID-19: yes (vs. no)	0.05 (0.02)	0.03
Seeking psychological intervention: yes (vs. no)	0.18 (0.02)	<0.001

**Table 3:** Model fit statistics of latent growth mixture models with 1–7 trajectory classes *.

Number of Classes	AIC	BIC	aBIC	*p* Value for LMR-LRT	Entropy	Proportion for Latent Classes (%)
1 class	263,472.560	263,558.820	263,523.863	/	/	/
2 classes	262,405.602	262,515.388	262,470.896	<0.001	0.601	52.0/48.0
3 classes	260,174.652	260,307.963	260,253.938	<0.001	0.774	55.4/38.3/6.3
4 classes	258,529.998	258,686.835	258,623.276	<0.001	0.783	44.4/31.2/18.1/6.3
5 classes	256,087.821	256,268.183	256,195.091	<0.001	0.850	41.5/31.1/18.0/6.8/2.7
6 classes	255,050.510	255,254.397	255,171.771	<0.001	0.849	32.3/31.9/18.9/7.5/6.8/2.7
7 classes	254,905.117	255,132.530	255,040.370	<0.001	0.803	35.2/26.3/18.0/6.9/6.8/4.2/2.7

* AIC = Akaike information criterion; BIC = Bayesian information criterion; LMR-LRT = Lo–Mendell–Rubin likelihood ratio test.

**Table 4:** Demographic and epidemic-related characteristics of the four latent trajectory classes.

Factors	Lowest–Great Increase (N = 1191)	Moderate Low–Slight Increase (N = 8347)	Moderate High–Slight Decrease (N = 3405)	Highest–Great Decrease (N = 5861)
Gender				
Male	570 (47.9) ^b,c^	3899 (46.7) ^b,c^	1492 (43.8)	2597 (44.3)
Female	621 (52.1)	4448 (53.3)	1913 (56.2)	3264 (55.7)
Age group (years)				
18–39	804 (67.5) ^c^	5703 (68.3) ^c^	2269 (66.6) ^c^	3588 (61.2)
≥40	387 (32.5)	2644 (31.7)	1136 (33.4)	2273 (38.8)
Living area				
Urban	1105 (92.8)	7787 (93.3) ^c^	3191 (93.7)	5516 (94.1)
Rural	86 (7.2)	560 (6.7)	214 (6.3)	345 (5.9)
Educational level				
College school or higher	929 (78.0) ^a,b,c^	6961 (83.4) ^c^	2874 (84.4) ^c^	4725 (80.6)
Lower than college school	262 (22.0)	1386 (16.6)	531 (15.6)	1136 (19.4)
Marital status				
Married	956 (80.3)	6531 (78.2)	2642 (77.6) ^c^	4654 (79.4)
Unmarried	235 (19.7)	1816 (21.8)	763 (22.4)	1207 (20.6)
Family income level (CNY)				
0–4999	295 (24.8) ^b^	1896 (22.7) ^b^	695 (20.4) ^c^	1300 (22.2)
≥5000	896 (75.2)	6451 (77.3)	2710 (79.6)	4561 (77.8)
COVID-19 patients or close contacts				
Yes	15 (1.3)	139 (1.7) ^b^	39 (1.1)	81 (1.4)
No	1176 (98.7)	8208 (98.3)	3366 (98.9)	5780 (98.6)
Engaged in work related to COVID-19				
Yes	474 (39.8) ^b,c^	3122 (37.4)	1231 (36.2)	2104 (35.9)
No	717 (60.2)	5225 (62.6)	2174 (63.8)	3757 (64.1)
Quarantine				
Yes	492 (41.3) ^a,b,c^	2947 (35.3) ^c^	1190 (34.9) ^c^	1751 (29.9)
No	699 (58.7)	5400 (64.7)	2215 (65.1)	4110 (70.1)
Living in places severely affected by COVID-19				
Yes	347 (29.1)	2578 (30.9) ^b,c^	933 (27.4)	1598 (27.3)
No	844 (70.9)	5769 (69.1)	2472 (72.6)	4263 (72.7)
Increases in workload due to COVID-19				
Yes	613 (51.5) ^b,c^	4295 (51.5) ^b,c^	1527 (44.8) ^c^	2364 (40.3)
No	578 (48.5)	4052 (48.5)	1878 (55.2)	3497 (59.7)
Unemployment due to COVID-19				
Yes	215 (18.1) ^a,b,c^	1266 (15.2) ^b,c^	379 (11.1)	698 (11.9)
No	976 (81.9)	7081 (84.8)	3026 (88.9)	5163 (88.1)
Seeking psychological consultation				
Yes	272 (22.8) ^a,b,c^	1363 (16.3) ^b,c^	424 (12.5) ^c^	561 (9.6)
No	919 (77.2)	6984 (83.7)	2981 (87.5)	5300 (90.4)

^a^: *p* < 0.05 for chi-squared tests for proportion differences compared with the “moderate low–slight increase” trajectory class; ^b^: *p* < 0.05 for chi-squared tests for proportion differences compared with the “moderate high–slight decrease” trajectory class; ^c^: *p* < 0.05 for chi-squared tests for proportion differences compared with the “highest–great decrease” trajectory class.

**Table 5:** Association between trajectory class membership of perceived accessibility to DMHSs and long-term positives of mental health symptoms during the COVID-19 pandemic.

Trajectory Class of Perceived Accessibility to DMHSs during COVID-19	n/N (%) of Mental Health Symptoms from Survey 1 (N = 16,508)	n/N (%) of Mental Health Symptoms from Survey 2 (N = 12,788)	n/N (%) of Mental Health Symptoms from Survey 3 (N = 13,175)	AOR (95% CI) *	*p* Value	Rate Decrease from Survey 1 to Survey 3 (% (95% CI))
**Any mental health symptoms**						
Lowest– great increase	733/1186 (61.8)	463/828 (55.9)	342/736 (46.5)	2.75 (2.47–3.05)	<0.001	15.3 (10.7–19.9)
Moderate low–slight increase	3890/6907 (56.3)	3070/5778 (53.1)	2746/6024 (45.6)	2.56 (2.41–2.72)	<0.001	10.7 (9.0–12.5)
Moderate high–slight decrease	1479/3389 (43.6)	946/2141 (44.2)	830/2281 (36.4)	1.79 (1.66–1.93)	<0.001	7.3 (4.6–9.9)
Highest–great decrease	1414/5026 (28.1)	1207/4041 (29.9)	993/4134 (24.0)	Reference	Reference	4.1 (2.3–5.9)
**Depression**						
Lowest– great increase	536/1186 (45.2)	379/828 (45.8)	251/736 (34.1)	2.92 (2.61–3.27)	<0.001	11.1 (6.5–15.6)
Moderate low–slight increase	2609/6907 (37.8)	2275/5778 (39.4)	1999/6024 (33.2)	2.52 (2.36–2.70)	<0.001	4.6 (3.0–6.3)
Moderate high–slight decrease	902/3389 (26.6)	670/2141 (31.3)	562/2281 (24.6)	1.77 (1.62–1.92)	<0.001	2.0 (−0.4–4.3)
Highest–great decrease	786/5026 (15.6)	792/4041 (19.6)	632/4134 (15.3)	Reference	Reference	0.4 (−1.2–1.9)
**Anxiety**						
Lowest– great increase	603/1186 (50.8)	363/828 (43.8)	230/736 (31.3)	3.01 (2.70–3.36)	<0.001	19.6 (15.1–24.0)
Moderate low–slight increase	2996/6907 (43.4)	2224/5778 (38.5)	1847/6024 (30.7)	2.64 (2.47–2.83)	<0.001	12.7 (11.1–14.4)
Moderate high–slight decrease	1037/3389 (30.6)	645/2141 (30.1)	501/2281 (22.0)	1.78 (1.64–1.94)	<0.001	8.6 (6.3–10.9)
Highest–great decrease	937/5026 (18.6)	758/4041 (18.8)	551/4134 (13.3)	Reference	Reference	5.3 (3.8–6.8)
**Insomnia**						
Lowest– great increase	521/1186 (43.9)	379/828 (45.8)	272/736 (37.0)	2.58 (2.31–2.88)	<0.001	7.0 (2.4–11.5)
Moderate low–slight increase	2589/6907 (37.5)	2408/5778 (41.7)	2123/6024 (35.2)	2.29 (2.14–2.45)	<0.001	2.2 (0.6–3.9)
Moderate high–slight decrease	924/3389 (27.3)	704/2141 (32.9)	624/2281 (27.4)	1.63 (1.50–1.78)	<0.001	−0.1 (−2.5–2.3)
Highest–great decrease	843/5026 (16.8)	874/4041 (21.6)	763/4134 (18.5)	Reference	Reference	−1.7 (−3.3–0.1)

* Values are from multivariable generalized estimating equation models adjusted for gender, age group, living area, marital status, educational level, history of chronic diseases, history of mental disorders and family history of mental disorders, being COVID-19 patients or having family members with the disease, engaging in COVID-19-related work, quarantine experiences, living in places severely hit by COVID-19, seeking psychological consultation, increases in workload due to COVID-19, unemployment due to COVID-19, history of sleep problems, history of smoking, and history of alcohol abuse. Rate decrease is calculated by subtracting rate of mental health problems in Survey 3 from the rate in Survey 1.

**Table 6:** Association between trajectory class of perceived accessibility to DMHSs and PHQ-9, GAD-7, and ISI scores during the COVID-19 pandemic.

Trajectory Class of Perceived Accessibility to DMHSs during COVID-19	Median (IQR) of Mental Health Scores from Survey 1	Median (IQR) of Mental Health Scores from Survey 2	Median (IQR) of Mental Health Scores from Survey 3	B (SE) for Main Effect *	*p* Value	B (SE) for Interaction with Time *	*p* Value
Depression							
Lowest–great increase	3.00 (0.00–9.00)	3.00 (0.00–10.00)	0.00 (0.00–9.00)	2.67 (0.12)	<0.001	−0.05 (0.01)	<0.001
Moderate low–slight increase	2.00 (0.00–9.00)	1.00 (0.00–9.00)	0.00 (0.00–8.00)	1.71 (0.06)	<0.001	−0.03 (0.00)	<0.001
Moderate high–slight decrease	0.00 (0.00–5.00)	0.00 (0.00–7.00)	0.00 (0.00–4.00)	0.84 (0.08)	<0.001	−0.01 (0.00)	0.10
Highest–great decrease	0.00 (0.00–1.00)	0.00 (0.00–2.00)	0.00 (0.00–1.00)	Reference	Reference	Reference	Reference
Anxiety							
Lowest–great increase	5.00 (0.00–9.00)	2.00 (0.00–8.00)	0.00 (0.00–7.00)	2.61 (0.11)	<0.001	−0.10 (0.01)	<0.001
Moderate low–slight increase	3.00 (0.00–7.00)	1.00 (0.00–7.00)	0.00 (0.00–7.00)	1.71 (0.05)	<0.001	−0.06 (0.00)	<0.001
Moderate high–slight decrease	1.00 (0.00–6.00)	0.00 (0.00–6.00)	0.00 (0.00–3.00)	0.85 (0.07)	<0.001	−0.03 (0.00)	<0.001
Highest–great decrease	0.00 (0.00–3.00)	0.00 (0.00–2.00)	0.00 (0.00–0.00)	Reference	Reference	Reference	Reference
Insomnia							
Lowest–great increase	6.00 (1.00–11.00)	7.00 (2.00–12.00)	4.00 (1.00–10.00)	2.36 (0.12)	<0.001	−0.05 (0.01)	<0.001
Moderate low–slight increase	5.00 (2.00–10.00)	6.00 (2.00–11.00)	5.00 (1.00–9.00)	2.00 (0.07)	<0.001	−0.04 (0.00)	<0.001
Moderate high–slight decrease	4.00 (1.00–8.00)	4.00 (1.00–9.00)	4.00 (1.00–8.00)	1.09 (0.08)	<0.001	−0.01 (0.01)	0.23
Highest–great decrease	2.00 (0.00–6.00)	2.00 (0.00–7.00)	1.00 (0.00–6.00)	Reference	Reference	Reference	Reference

* Values are from multivariable generalized mixed linear models adjusted for fixed effects for gender, age group, living area, marital status, educational level, history of chronic diseases, history of mental disorders and family history of mental disorders, being COVID-19 patients or close contacts, engaging in COVID-19-related work, quarantine experiences, living in places severely hit by COVID-19, seeking psychological consultation, increases in workload due to COVID-19, unemployment due to COVID-19, history of sleep problems, history of smoking, and history of alcohol and their interactions with time.

## Data Availability

The corresponding authors have full access to all the data in the study and take responsibility for the integrity of the data and the accuracy of the data analyses.

## Supplementary Materials

The following supporting information is available online at https://www.mdpi.com/article/10.3390/ijerph19063593/s1, Table S1: Multinomial logistic regression of predictors for trajectory class membership of perceived accessibility to DMHSs; Figure S1: Changes in proportion of actual DMHSs usage from before to one year after COVID-19 outbreak; Table S2: Demographic characteristics of full baseline sample and longitudinal sample.
