# Supplementary material for: Accessibility to Digital Mental Health Services among the General Public throughout COVID-19: Trajectories, Influencing Factors and Association with Long-Term Mental Health Symptoms
Source: Int J Environ Res Public Health. 2022 Mar 17;19(6):3593. doi: 10.3390/ijerph19063593 (PMC8955845; doi:10.3390/ijerph19063593)
Supplement: Supplementary file 1 [file ijerph-19-03593-s001.zip › ijerph-1627463-supplementary.pdf]

Table S1. Multinomial logistic regression of predictors for trajectory class membership of perceived accessibility to DMHSs.

**Factors**	**Odds ratio [95%CI] for lowest-great increase vs. moderate low-slight increase**	**Odds ratio [95%CI] for lowest-great increase vs. moderate high-slight decrease**	**Odds ratio [95%CI] for lowest-great increase vs. highest-great decrease**	**Odds ratio [95%CI] for moderate low-slight increase vs. moderate high-slight decrease**	**Odds ratio [95%CI] for moderate low-slight increase vs. highest-great decrease**	**Odds ratio [95%CI] for moderate high-slight decrease vs. highest-great decrease**
**Gender: male (vs. female)**	1.05 [0.93-1.18]	1.18 [1.03-1.34] *	1.15 [1.02 - 1.31] *	1.19 [1.07-1.32] *	1.10 [1.03-1.18] *	0.98 [0.90-1.07]
**Age group (years): 18-39 (vs. ⩾40)**	0.96 [0.85-1.10]	1.04 [0.90-1.20]	1.32 [1.15-1.50] *	1.08 [0.99-1.18]	1.37 [1.27-1.47] *	1.27 [1.16-1.38] *
**Living area: urban (vs. rural)**	0.92 [0.73-1.17]	0.86 [0.67-1.12]	0.80 [0.63-1.03]	0.93 [0.79-1.10]	0.87 [0.76 -1.00] *	0.93 [0.78-1.11]
**Educational level: college school or higher (vs. lower than college school)**	0.71 [0.61-0.82] *	0.66 [0.56-0.77] *	0.85 [0.73-0.99] *	0.93 [0.83-1.04]	1.21 [1.11-1.32] *	1.30 [1.16-1.46] *
**Marital status: married (vs. unmarried)**	1.13 [0.97-1.32]	1.18 [1.00-1.38]	1.06 [0.90-1.23]	1.04 [0.94-1.14]	0.93 [0.86-1.01]	0.90 [0.81-1.00] *
**Income level (yuan): 0-4999 (vs.⩾5000)**	1.12 [0.97-1.29]	1.28 [1.10-1.50] *	1.16 [1.00-1.34]	1.15 [1.04-1.26] *	1.03 [0.95 - 1.12]	0.90 [0.81-1.00] *
**COVID-19 patients or close contacts: yes (vs. no)**	0.75 [0.44-1.29]	1.10 [0.61-2.00]	0.91 [0.52-1.59]	1.46 [1.02-2.09] *	1.21 [0.92-1.59]	0.83 [0.56-1.21]
**Engaged in work related to COVID-19: yes (vs. no)**	1.11 [0.98-1.25]	1.17 [1.02-1.34] *	1.18 [1.04-1.34] *	1.06 [0.97-1.15]	1.07 [1.00-1.14]	1.01 [0.93-1.10]
**Quarantine: yes (vs. no)**	1.29 [1.14-1.46] *	1.31 [1.15-1.50] *	1.65 [1.45-1.88] *	1.02 [0.93-1.10]	1.28 [0.19-1.38] *	1.26 [1.15-1.38] *
**Living in places severely affected by COVID-19: yes (vs. no)**	0.92 [0.81-1.05]	1.09 [0.94-1.26]	1.10 [0.96-1.26]	1.18 [1.08-1.29] *	1.19 [1.11-1.28] *	1.01 [0.92-1.11]
**Increases in workload due to COVID-19: yes (vs. no)**	1.00 [0.89-1.13]	1.30 [1.14-1.49] *	1.57 [1.38-1.78] *	1.30 [1.20-1.41] *	1.57 [1.47 – 1.68] *	1.20 [1.10-1.31] *
**Unemployment due to COVID-19: yes (vs. no)**	1.23 [1.05-1.45] *	1.76 [1.47-2.11] *	1.63 [1.38-1.93] *	1.43 [1.26-1.61] *	1.32 [1.20-1.46] *]	0.93 [0.81-1.06]
**Seeking psychological consultation: yes (vs. no)**	1.52 [1.31-1.76] *	2.08 [1.76-2.47] *	2.80 [2.38-3.28] *	1.37 [1.22-1.54] *	1.84 [1.66-2.05] *	1.34 [1.18-1.54] *

* $P < 0.05$ for odds ratio from multinomial logistic regression.

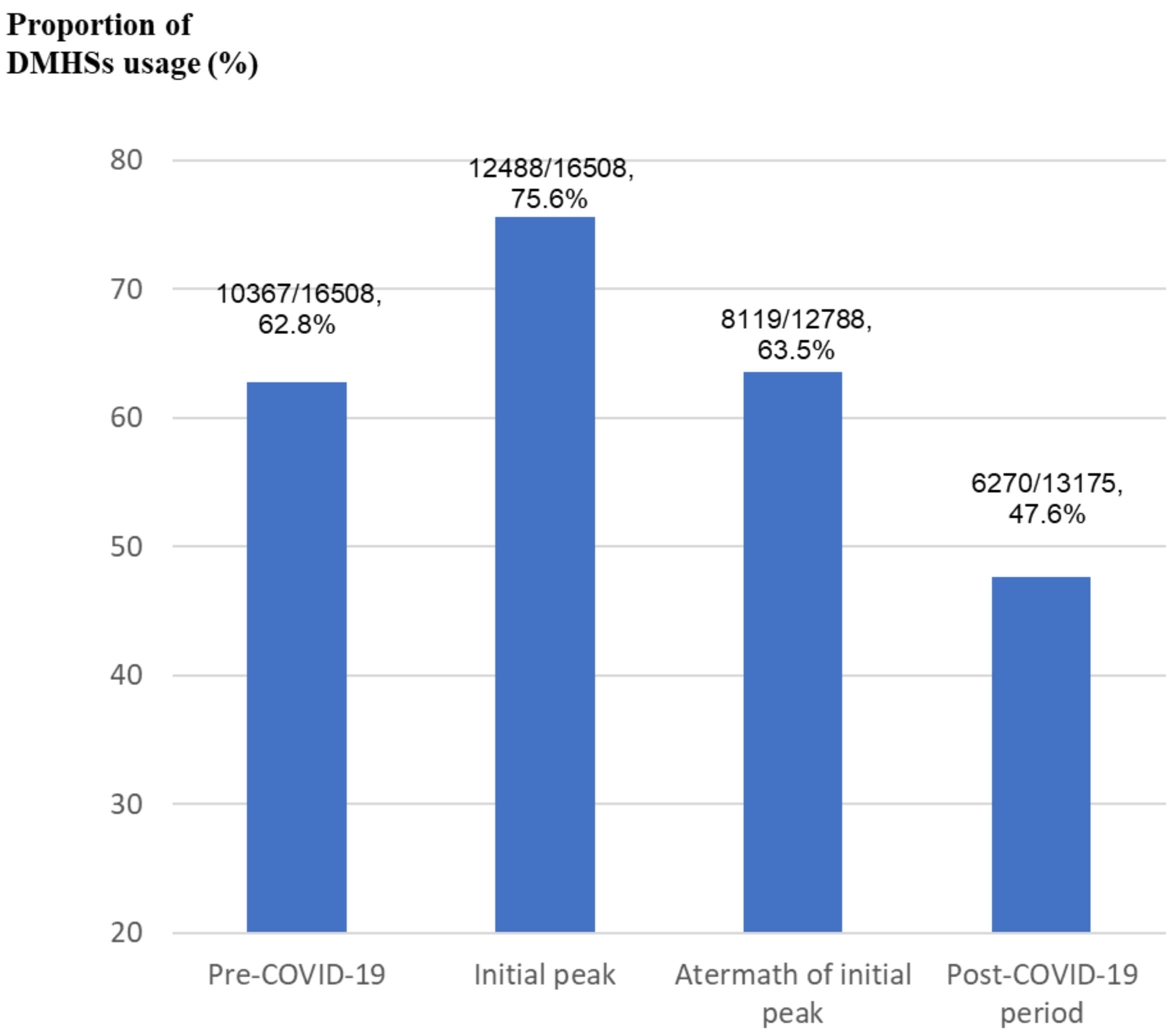

Figure S1. Changes in proportion of actual DMHSs usage from before to one year after COVID-19 outbreak.

Table S2. Demographic characteristics of full baseline sample and longitudinal sample.

Factors	Full baseline sample (n=56679)	Longitudinal sample (n = 18804)
**Gender**		
Male	27149(47.9)	8558(45.5)
Female	29530(52.1)	10246(54.5)
**Age group (years)**		
18-39	39468(69.6)	12364(65.8)
⩾40	17211(30.4)	6440(34.2)
**Living area**		
Urban	52839(93.2)	17599(93.6)
Rural	3840(6.8)	1205(6.4)
**Educational level**		
College school or higher	47139(83.2)	15489(82.4)
Lower than college school	9540(16.8)	3315(17.6)
**Marital status**		
Married	43763(77.2)	14783(78.6)
Unmarried	12916(22.8)	4021(21.4)
**Family monthly income (yuan)**		
0-4999	13016(23.0)	4186(22.3)
⩾5000	43663(77.0)	14618(77.7)
**History of chronic diseases**		
Yes	3274(5.8)	1201(6.4)
No or unknown	53405(94.2)	17603(93.6)
**History of psychiatric disorders**		
Yes	161(0.3)	122(0.6)
No or unknown	56518(99.7)	18682(99.4)
**Family history of psychiatric disorders**		
Yes	396(0.7)	235(1.2)
No or unknown	56283(99.3)	18569(98.8)
